# The use of in-strip digestion for fast proteomic analysis on tear fluid from dry eye patients

**DOI:** 10.1371/journal.pone.0200702

**Published:** 2018-08-03

**Authors:** Zhu Huang, Chi-Xin Du, Xiao-Dong Pan

**Affiliations:** 1 Department of Ophthalmology, the First Affiliated Hospital, College of Medicine, Zhejiang University, Hangzhou, China; 2 Department of Ophthalmology, the Fourth Affiliated Hospital, Zhejiang University School of Medicine, Yiwu, China; 3 Zhejiang Provincial Center for Disease Control and Prevention, Hangzhou, China; Università della Calabria, ITALY

## Abstract

Tear is an accessible fluid for exploring biomarkers of dry eye disease. This study describes a fast proteomic method by LC-Q-orbitrap-MS analysis with in-strip digestion and investigates the tear proteome of dry eye patients. Schirmer’s strips were used for collection of tear fluid from patients. These strips were cut into pieces and directly digested with trypsin before mass spectrometry analysis. The data showed that more than 50 proteins were found in tear fluid from dry eye patients. Gene Ontology (GO) annotation showed that most of proteins were transfer/carrier proteins, hydrolyses, enzyme modulators and signaling molecules. Targeted proteomics strategy revealed that 18 proteins were differentially expressed in dry eye patients. Furthermore, it was showed that the common post-translational modification in tear proteins is deamidation of Asn.

## Introduction

Human tear is a complex fluid comprised of secretions from different sources including the lacrimal gland, goblet cells, cornea, and vascular sources. The protein concentration in tear can reach 8–10 μg/μL and is increasingly investigated for exploring biomarkers of eye diseases [1, 2]. For example, ocular surface inflammation can be marked by well-known inflammation-related proteins (S100 A8 and S100 A9). Lactoferrin a major iron-binding protein with both immunomodulatory and antimicrobial activities [3, 4] is associated with the aqueous-deficient dry eye [5, 6].

The recent developments in proteomics and mass spectrometry have improved our understanding of proteins or peptides in the tears. Large amounts of tear proteins (close to 2000) have been revealed in human body [6, 7]. The endogenous peptides in human reflex tears were also identified. Hayakawa et al. [8] have analyzed and identified 30 peptides derived from two different proteins (proline-rich protein 4 and polymeric immunoglobulin receptor. Azkargorta et al. [9] have identified 234 peptides derived from 25 proteins in a human basal tear sample. MS-based proteomic analysis of tear fluid can reveal basic biological information for many ocular diseases, such as diabetic retinopathy, keratoconus, thyroid eye disease, vernal keratoconjunctivitis and primary open angle glaucoma [10–14]. However, despite the potential of the tear as a source of noninvasive clinic samples, mass spectrometry proteomic is not yet widely used in routine clinical.

Mass spectrometry (MS) is a fundamental and versatile technique for analyte test in biological samples due to its speed, specificity, sensitivity and throughput [15]. The current modes for biomarker quantification based on MS are selected/multiple reaction monitoring (SRM or MRM) performed typically on a triple quadrupole mass spectrometer and parallel reaction monitoring (PRM) performed on a hybrid quadrupole-orbitrap [16] or a quadrupole time-of-flight [17] MS. For example, Tong et al. [18] quantified of 47 human tear proteins using high resolution multiple reaction monitoring (HR-MRM) of Triple TOF-MS. You et al. [19] have determined human tear lactoferrin using MRM technique with stable-isotopic labeling. However, it should be noted that unlike clinical immunoassays, MS-based analyses of proteins/peptides from biological fluids usually involve complicated pretreatments, such as analyte extraction and tryptic digestion [20].

Usually, sample preparation plays an important role in reducing systematic and random analytical errors, which can ensure accurate detection and reproducible removal of interferences [21]. Proteomic test does not usually involve protein purification via performing sophisticated 2-D liquid chromatography or gel electrophoresis. Most of targeted protein content can be separated by precipitating it with solvents, such as ethanol, acetone, methanol, and acetonitrile (ACN) [21, 22]. In tear proteomic, tear fluid is usually collected by Schirmer’s strip and the proteins are washed and separated from strips for later treatments [7, 19, 23–25]. However, more steps in sample preparation will lead to more uncertainty of analysis results, especially for the sample with small volume.

In this study, we developed a fast pretreatment method for proteomic analysis by liquid chromatography couple quadrupole-orbitrap mass spectrometry (LC-Q-Orbitrap-MS) and investigated the proteins in tears from moderate dry eye patients. The tear fluid was collected by Schirmer’s strip. Proteins in strips were directly digested without further separation, which we named “in-strip” digestion. The digested solution was then cleaned by C18 column (ZipTip) before instrumental analysis.

## Materials and methods

### Chemicals and reagents

Dithiotheritol (DTT), iodoacetamide (IA), ammonium bicarbonate (NH_4_HCO_3_) and hydrochloric acid (HCl, 37%) were obtained from Sigma–Aldrich (St. Louis, MO, USA). Formic acid (FA) and acetonitrile (ACN) were purchased from Merck (Darmstadt, Germany). All the reagents used were analytical or HPLC grade. Sequencing grade modified trypsin was from Shanghai Yaxin Biotechnology Co., Ltd (Shanghai, China). All chemical agents were prepared using ultrapure water and without further purification. Ultrapure water was obtained by a Milli-Q Gradient A10 water purification system (Millipore, Bedford, MA, USA) during all the experiments.

### Human tear samples

**Fig 1:**
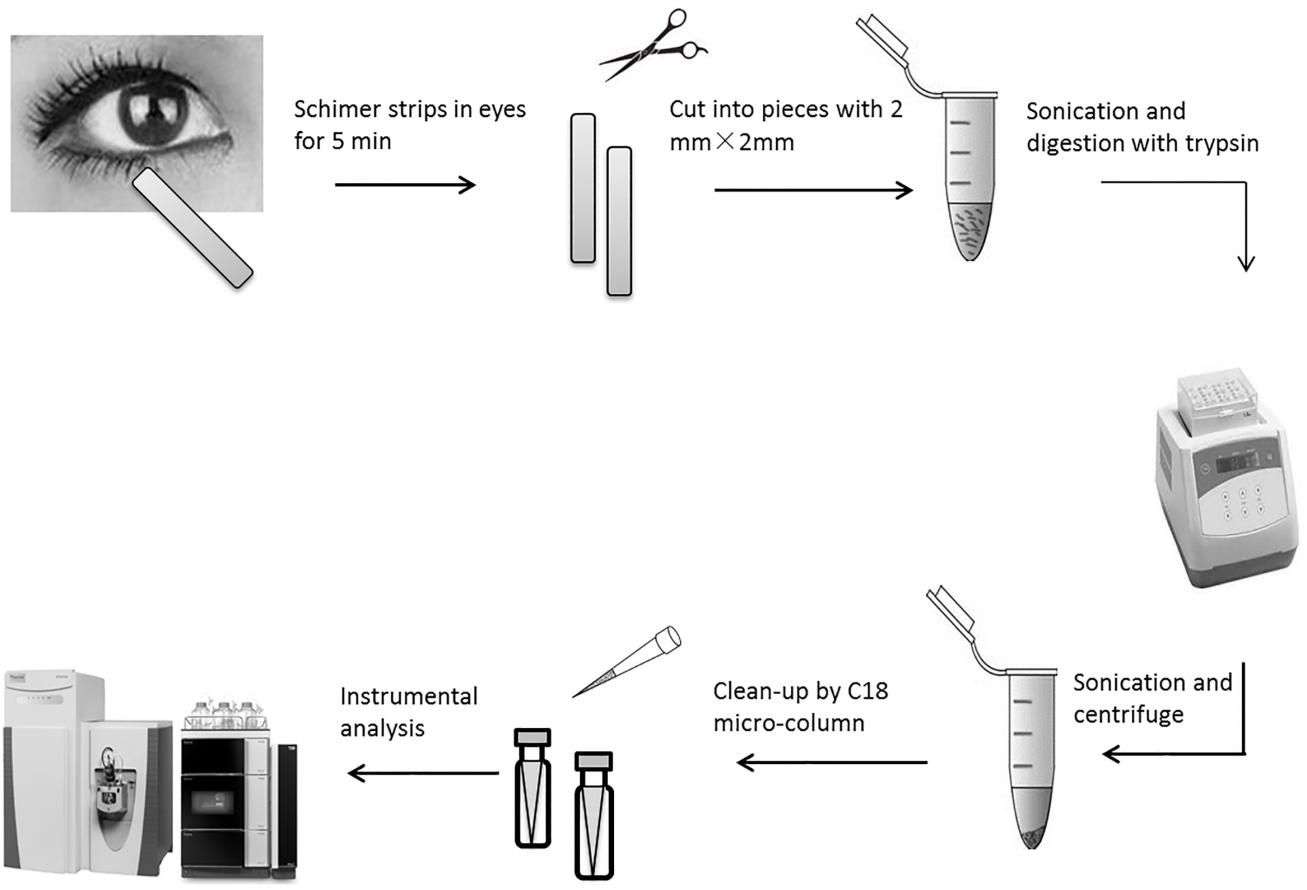
The workflow of the sample preparation for tear proteomic analysis.

The tear fluid was from volunteers with moderate dry eye syndrome. All experiments on the human eye were approved by Human Research Ethics Committee of the First Affiliated Hospital, College of Medicine, Zhejiang University and performed in accordance with the relevant guidelines. We confirmed that informed consent in writing was obtained from all subjects for usage of human tear. Diagnosis of dry eye and the grading of the severity is based on various clinical parameters such as Schirmer’s test ≤10 mm for 5 min, without anesthesia), tear breakup time (TBUT) (≤10 s), corneal and conjunctival staining score based on Dry Eye Work Shop study (DEWS) as well as using MacMonnies questionnaire [26]. The workflow of sample test was shown in Fig 1. Tear samples were collected by Schirmer’s type I tear test without using local anesthesia (Tear from volunteers were collected from October 5 to October 20, 2017). The Schirmer strips were inserted for 5 min in the lower eyelid in the standard fashion in both eyes by the same subject, and the strip was filled with tears. The wet parts of strips were collected and cut into pieces with 2 mm × 2 mm. The pieces of strips were then placed in a 2-mL centrifuge tube and immediately frozen at -80 °C until analysis. These samples were analyzed in December 1, 2017. Samples were carefully marked that authors had access to information that could identify individual participants during or after data collection. All experiments on the human eye were approved by Human Research Ethics Committee of the First Affiliated Hospital, College of Medicine, Zhejiang University and performed in accordance with the relevant guidelines. We confirmed that informed consent was obtained from all subjects for usage of human tear.

### Sample preparation

The tube with strip pieces were mixed with 100 μL 500 mM NH_4_HCO_3_ and 275 μL deionized water. The mixture was sonicated for 10 min at maximum intensity and heated at 120 °C for 5 min. Then, 10 μL 50 mM DTT solution was added to the mixtures and reduced in 40 °C water bath for 30 min at this stage. Subsequently, an alkylation was performed by adding 10 μL of 150 mM IAA in the dark for 30 min at room temperature. Immediately prior to the incubation, 100 μL of 500 mM NH_4_HCO_3_ and 10 μL of 400 μg/mL trypsin (freshly prepared) were added and incubated 12 h at 40 °C. The reaction was terminated by addition of 5 μL formic acid (FA). After that, the mixture was sonicated for 10 min at maximum intensity and centrifuged at 13000 g for 10 min. The supernatant was desalted and eluted with ZipTip C18 cartridges (Millipore, Bedford, MA) [27]. Briefly, ZipTips were conditioned with 20 μL ACN and equilibrated with 20 μL 0.1% FA. The digested solutions (containing peptides) were then loaded onto the ZipTip and washed with 10 μL 0.1% FA (repeated washing for five times). Finally, peptides were eluted twice with 10 μL 50% ACN containing 0.1% FA and diluted with 50% ACN containing 0.1% FA to 40 μL.

### Chromatographic and MS conditions

UHPLC system consisting of pumps, an autosampler and a column oven was used in this test (Vanquish, Thermo Scientific, San Jose, CA, USA). Chromatographic separation was performed on a 2.1 × 100 mm, 1.7 μm, BEH 300 C18 column (Waters Corporation, MA, USA). The column temperature was set at 35°C. Mobile phase A consisted of 0.1% formic acid in water, and mobile phase B consisted of 0.1% formic acid in acetonitrile. Samples were separated by gradient elution using the following program: 0 min 3% B; 0–0.8 min 3% B; 0.8–30 min 20% B; 30–50 min 70% B; 50–55 min 100% B; 55–57 min 100% B; 57–58 min 3% B, 58–60 min 3% B. The flow rate was 0.3 mL/min. The sample injection volume was 5 μL.

For HR-MRM experiments, UPLC system was coupled to a Q-Orbitrap-MS (QE Extrative, Thermo Fisher Scientific, San Jose, CA, USA). The mass spectrometer was operated in positive mode with a electrospray voltage 3.2 kV, capillary temperature 350 °C, vaporizer temperature 250 °C, sheath gas (N_2_) 40 arbitrary units (arb), auxiliary gas (N_2_) 15 (arb), and S-Lens RF level at 50 (arb). Data dependent acquisition (DDA) was adopted for peptide identification, namely Full MS/dd-MS^2^ in instrument parameters. For a Full MS scan, the selected scan range was from *m/z* 100 to 1500 and the resolution was 35 000 (FWHM at *m/z* 200), while the automatic gain control (AGC) target (the number of ions to fill C-Trap) was set to 1.0e6 with a maximum injection time (IT) of 100 ms. For the dd-MS^2^ scan, fragmentation mass spectra were set at a mass resolving power of 17 500 FWHM with a quadrupole isolation window of 0.4 Da for precursor ions. Other MS parameters for the dd-MS^2^ scan were applied as follows: AGC target 2.0e5, maximum IT 50 ms, underfill ratio 1.0%, intensity threshold 4.0e4, exclude isotopes ‘on’, and dynamic exclusion 10.0 s. The instrument was calibrated in positive mode every 7 days using the Pierce LTQ Velos ESI positive-ion calibration solutions (containing caffeine, the tetrapeptide MRFA and a mixture of fluorinated phosphazines ultramark 1621).

### Data analysis

The instrumental system was controlled using the software packages Xcalibur 3.0, Chromeleon MS Link 2.14 and Q-Exactive Tune 2.3 (all Thermo Scientific). Xcalibur software was used for data acquisition and processing. For identification analytical runs, full MS/dd-MS^2^ scan was adopted. The data search was perfomed by the software of Proteome Discoverer 2.1 coupled with SEQUEST search engine (Thermo Fisher Scientific, Waltham, MA, USA) for peptide identification. Relative quantification of identified proteins was made by MaxQuant software version 1.5.3.30 (http://www.maxquant.org). Human protein information was downloaded from human UniProt/Swiss-Prot version 2017_09 (555,426 entries). False discovery rate (FDR) analysis in software was performed and FDR < 1% was set for both peptide and protein identification. Candidate proteins were inspected by gene ontology (GO) analysis (http://www.geneontology.org/). Interaction analysis was realized by use of STRING version 10 (http://string-db.org) considering a medium confidence score of 0.4 for interactions. Data of protein quantification were analyzed by the SPSS version 16.0 (SPSS, Chicago, IL). Student’s t tests were performed and a *P* value less than 0.05 was considered significant in our study.

## Results and discussion

### Optimization of sample preparation

The method for tear collection is an important step which can affect results of tear analysis. Schirmer’s strip (Schirmer Type I test, without anesthesia) and glass capillary tubes are the two most popular tools for tear collection. As a standard clinical test, the Schirmer’s strip test is routinely used for diagnosis of dry eye syndrome. For proteomic analysis, previous study has proved that more proteins were identified by using Schirmer’s strips than glass capillary tubes [28]. Accordingly, most researchers adopted Schirmer’s strips for tear fluid collection, and extracted proteins from these strips for subsequent test [7, 19, 23–25]. In this study, we also used the Schirmer’s strip for tear collection. Subsequently, strips containing proteins were cut into small pieces and directly digested by trypsin (named in-strip digestion). Comparing with the method of protein exaction by organic solution, in-strip digestion owns fewer procedures and easier operations, which may avoid exogenous contamination and reduce matrix interferences. Our pre-experiment showed that more proteins were identified by in-strip digestion (86 proteins) than method of protein exaction (65 proteins). Furthermore, for extraction of digested peptides, we found that sonication before centrifuging can enhance the distribution of peptides in solution.

### Tear proteins and post-translational modifications (PTMs)

**Fig 2:**
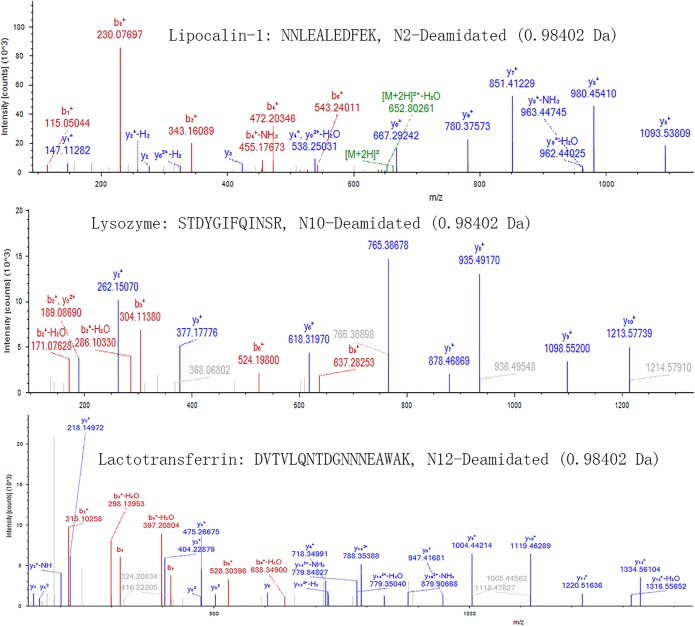
Mass spectra of deamidated peptides from lactotransferrin, lysozyme and lipocalin-1.

The data showed that 86 proteins were found by MS-based proteomic analysis (See S1 Table). Furthermore, post-translational modifications (PTMs) of proteins were investigated by software of Proteome Discoverer. The PTMs can affect various properties of proteins, including the enzymatic activity, protein interactions and subcellular location [27]. The current proteomic technologies allow large-scale analyses of many PTMs, such as methylation, acetylation, phosphorylation, ubiquitination, and glycosylation [29, 30]. In spite of the general interest in the effect of PTMs on the protein function, little has been done to characterize the large array of modifications in tear proteins. You et al. have attempted to characterize tear protein phosphorylation and glycosylation using 2DE and specific dyes [31]. They have presented the first experimental evidence of lipocalin and cystatin S glycosylation, and identified the tear dermcidin. The study has validated the presence of dermcidin in human tears using MRM and revealed the phosphorylation of nucleobinding. As shown in Table 1, modification of main proteins in tears was investigated by Proteome Discoverer software. We found that deamidation of Asn (N) appeared in lactotransferrin, lysozyme, Lipocalin-1, Ig gamma-1 chain C, prolactin-inducible protein, and etc. The ratios of deaminated Asn to normal Asn were all more than 20%. Some mass spectra of deamidated peptides from lactotransferrin, lysozyme and lipocalin-1 were shown in Fig 2. Deamidation is a possible regulator of protein–ligand and protein–protein interactions. It was reported that deamidation of Asn in complementarity determining region (CDR) of an antibody were linked to the loss of antibody binding affinity [32]. Furthermore, methylation of Lys/Arg (K/R) and acylation of Ser (S) were found in lipocalin-1 and lysozyme separately. Methylation has the function of regulation of gene expression. Acetylation plays the role of cellular localization and targeting signals, membrane tethering, and mediator of protein–protein interactions [33, 34]. Acetylation suggests a broader use of acetylation in cellular regulation mechanism. However, it should be noted that we did not explore the difference of PTMs between dry eye patients and normal persons due to technical limits.

**Table 1:** The post-translational modifications of some proteins in tear fluid.

Main protein	Number of identified peptides	Deamidated ratio of N	Methylation ratio of L	Methylation ratio of K	Acylation ratio of S
Lactotransferrin	34	42.9% (9/21)	0 (0/34)	0 (0/21)	0 (0/21)
Lysozyme	8	22.2% (2/9)	33.3% (1/3)	0 (0/3)	33.3% (2/6)
Lipocalin-1	5	66.7% (4/6)	12.5% (1/8)	25% (1/4)	0 (0/3)
Ig gamma-1 chain C	6	33.3% (1/3)	0 (0/4)	0 (0/5)	0 (0/8)
Zinc-alpha-2-glycoprotein	8	0 (0/2)	0 (0/4)	0 (0/4)	0 (0/3)
Prolactin-inducible protein	5	25% (1/4)	0 (0/3)	0 (0/3)	0 (0/1)
Keratin, type II cytoskeletal 1	9	0 (0/7)	0 (0/12)	0 (0/7)	0 (0/5)
Mammaglobin-B	4	0 (0/2)	0 (0/5)	0 (0/3)	0 (0/3)
Apolipoprotein A-I	7	0 (0/1)	0 (0/12)	0 (0/7)	0 (0/6)
Protein S100-A8	4	50% (1/2)	0 (0/3)	0 (0/3)	0 (0/1)
Protein S100-A9	3	0 (0/1)	0 (0/3)	0 (0/4)	0 (0/1)
Extracellular glycoprotein lacritin	4	100% (1/1)	0 (0/4)	0 (0/5)	0 (0/5)
Cystatin-S	3	0 (0/2)	0 (0/1)	0 (0/1)	0 (0/1)

### Comparison between dry eye patients and normal persons

Main proteins selected for comparison are involved in the inflammatory response (α-2-HS-glycoprotein, transferrin and orosomucoid 1), biosynthesis of IL-8 (apolipoprotein) and activation of the host immune response and the inflammatory response (clusterin, keratin 1, and C3). Table 2 shows the changes of main tear proteins between dry eye patients (*n* = 8) and normal group (*n* = 8). This analysis ascertained the differentially expressed profiles of 18 proteins (*P < 0*.*05*). Among these identified proteins, lactotransferrin (LTF), lysozyme (LYZ), lipocalin 1 (LCN1), zinc-alpha-2-glycoprotein (AZGP1), secretoglobin, family 2A, member 1 (SCGB2A1), deleted in malignant brain tumors 1 (DMBT1), lacritin (LACRT) and proline rich 4 (PRR4) were found to be significantly decreased in moderate dry eye patients. On the contrary, transferrin (TF), Keratin 1 (KRT1), polymeric immunoglobulin receptor (PIGR), Complement component 3 (C3), S100A8, S100A9, orosomucoid 1 (ORM1), Annexin A1 (ANXAI), Immunoglobulin J polypeptide (IGJ) and Heat shock 27kDa protein 1 (HSPB1) were found to be significantly increased in dry eye group. The results were similar with the report of Perumal et al. [24]

**Table 2:** Changes of main tear proteins from dry eye patients.

Accession	Gene Name	Protein	
P02768	ALB	Albumin	—
P02788	LTF	Lactotransferrin	downregulated
P61626	LYZ	Lysozyme	downregulated
P02787	TF	Transferrin	upregulation
P31025	LCN1	Lipocalin 1	downregulation
P25311	AZGP1	Zinc-alpha-2-glycoprotein	downregulation
P12273	PIP	Prolactin-induced protein	—
P04264	KRT1	Keratin 1	upregulation
O75556	SCGB2A1	Secretoglobin, family 2A, member 1	downregulated
P02647	APOA1	Apolipoprotein A-I	—
P01009	SERPINA1	Serpin peptidase inhibitor 1	—
P01833	PIGR	Polymeric immunoglobulin receptor	upregulation
P01024	C3	Complement component 3	upregulation
P05109	S100A8	S100 calcium binding protein A8	upregulation
P00738	HP	Haptoglobin	—
Q9UGM3	DMBT1	Deleted in malignant brain tumors 1	downregulated
P06702	S100A9	S100 calcium binding protein A9	upregulation
Q9GZZ8	LACRT	Lacritin	downregulated
P01036	CST4	Cystatin S	—
Q99935	PROL1	Proline rich, lacrimal 1	—
P02763	ORM1	Orosomucoid 1	upregulation
P13645	KRT10	Keratin 10	—
P35908	KRT2	Keratin 2	—
P10909	CLU	Clusterin	—
P04083	ANXA1	Annexin A1	upregulation
O95968	SCGB1D1	Secretoglobin, family 1D, member 1	—
P02790	HPX	Hemopexin	—
P01591	IGJ	Immunoglobulin J polypeptide	upregulation
P02765	AHSG	alpha-2-HS-glycoprotein	
P02749	APOH	Apolipoprotein H (beta-2-glycoprotein I)	
P04792	HSPB1	Heat shock 27kDa protein 1	upregulation
P09211	GSTP1	Glutathione S-transferase pi 1	—
Q4L180	FILIP1L	Filamin A interacting protein 1-like	—
A5A3E0	POTEF	POTE ankyrin domain family, member F	—
P01023	A2M	Alpha-2-macroglobulin	—
Q08188	TGM3	Transglutaminase 3	—
Q16378	PRR4	Proline rich 4 (lacrimal)	downregulated
Q8NI51	CTCFL	CCCTC-binding factor (zinc finger protein)-like	—

The findings indicate that proteins in the tear fluid of dry eye patients are possibly related to the host stress response. Lactoferrin is one of the components of the immune system of the body. It has antimicrobial activity and functions of antiparasitic, catalytic, immunomodulatory and anti-inflammatory. Lysozymes are usually produced from certain secretions, such as tears, saliva, human milk, and mucus. Tear lipocalin, one of major proteins in tears, can bind a variety of lipophilic molecules and certain proteins like lactoferrin and lysozyme. It has many functions in tears, such as regulation of tear viscosity, anti-inflammatory activity, endonuclease inactivation of viral DNA. Previous study also found the downregulation of lipophilin-1 in dry eye disease [5]. Proline-rich proteins (PRPs) are highly polymorphic and belong to a class of intrinsically unstructured proteins. Proline-rich proteins (PRPs) are highly polymorphic and belong to a class of intrinsically unstructured proteins. PRR4 was considered as a potential biomarker of dry eye syndrome [35].

### Functions of tear proteins

**Fig 3:**
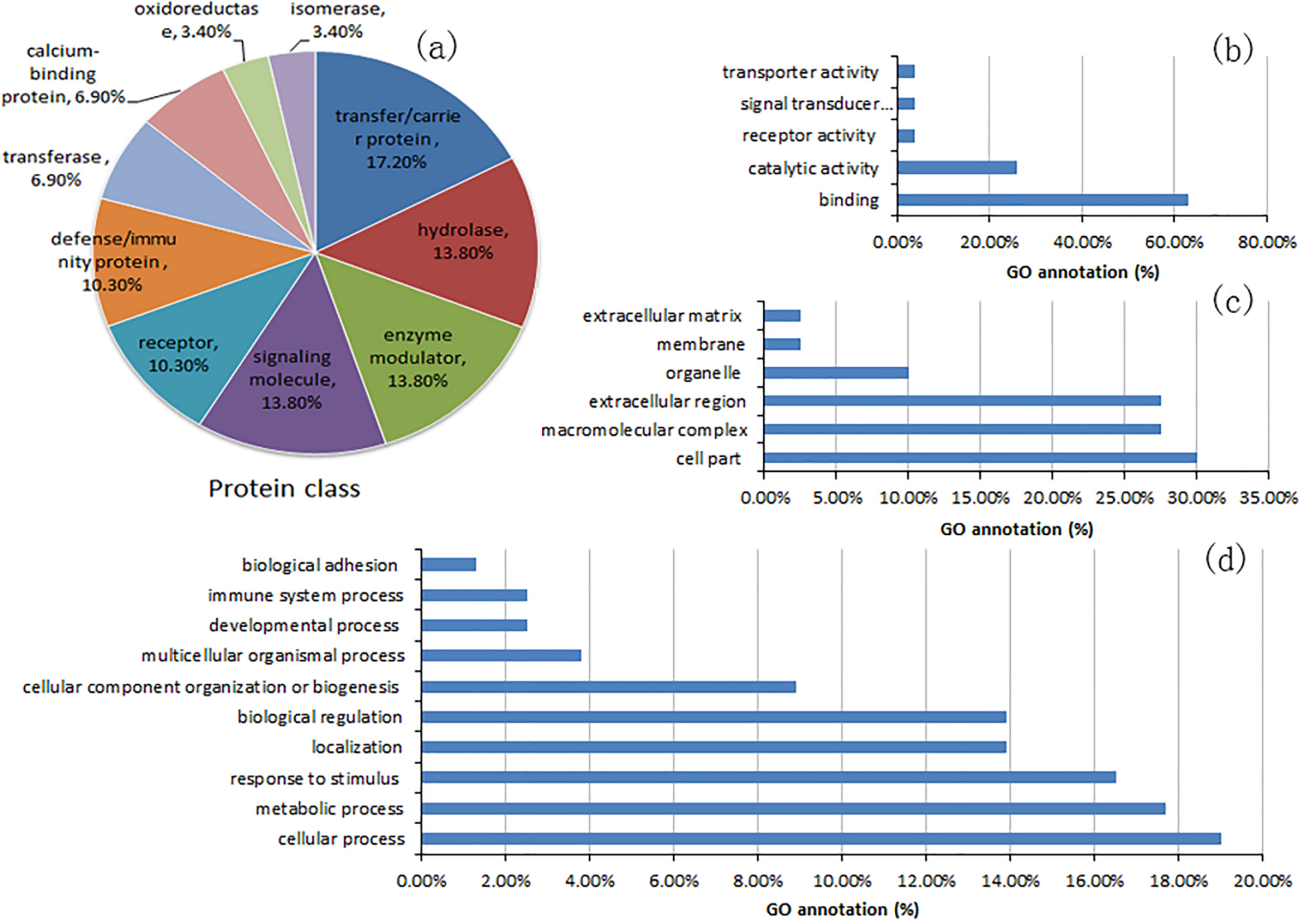
Localization and functional GO analysis of main tear proteins.

The proteins were further investigated by GO annotation. As shown in Fig 3, most of proteins were transfer/carrier protein, hydrolase, enzyme modulator and signaling molecule. The main function was binding and catalytic activity in molecular function, and cellular process and metabolic process in biological process. Previous reports have showed that proteins in tear fluid are usually classified into four types [7]: (1) proteins secreted by the main lacrimal gland, Meibomian glands, goblet cells and accessory lacrimal glands of the ocular surface, (2) ocular cell/tissue leakage products, (3) aberrant secretions and (4) foreign proteins.

**Fig 4:**
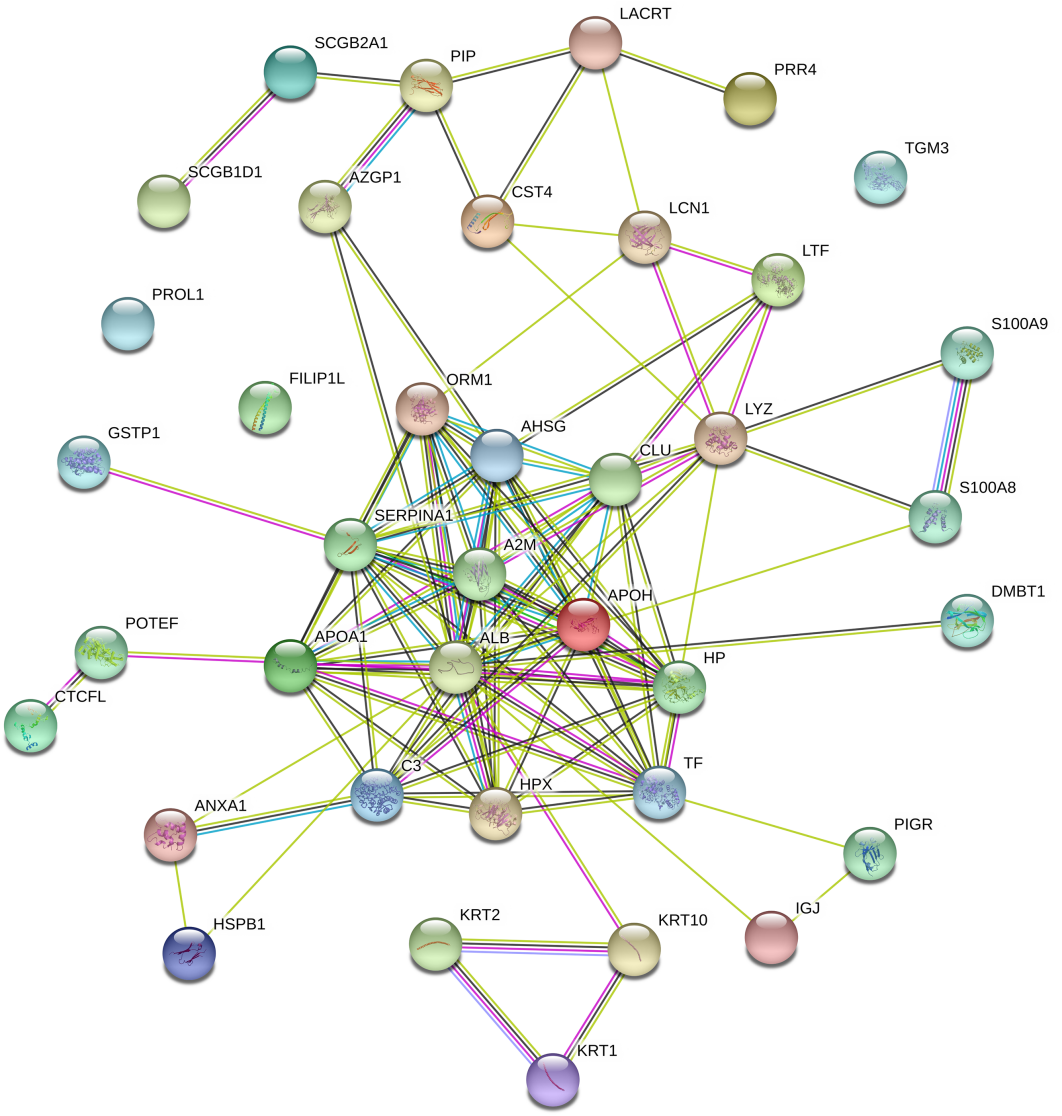
Interaction network of main tear proteins by STRING analysis.

To exploring the interaction of these proteins, we used database of STRING to show the mutual relations among identified proteins. Fig 4 shows the possible interaction, and line thickness indicates the strength of data support. We can observe that proteins of APOH (apolipoprotein H), SERPINA1 (serpin peptidase inhibitor), APOA1 (apolipoprotein A-I), TF (transferrins), AHSG (alpha-2-HS-glycoprotein), ORM1 (orosomucoid 1), ALB (serum albumin), CLU (clusterin), LYZ (lysozyme), HP (haptoglobin), A2M (alpha-2-macroglobulin) and HPX (hemopexin) have strong interaction relations. S100 A8 and S100 A9 were found to be associated with dry eye [36]. They are calcium- and zinc-binding protein which plays a prominent role in the regulation of inflammatory processes and immune response. We observed that S100 A9 and S100 A8 have interacted relation with LYZ (lysozyme) and CLU (clusterin).

## Conclusion

The method of LC-Q-orbitrap-MS analysis with in-strip digestion can be applied in proteins test in human tear fluid. The data showed that 18 proteins were differentially expressed between moderate dye eye patients and normal persons. Furthermore, it indicates that deamidation of Asn was the common post-translational modification in tear proteins. The outcomes of the identification of these proteins can provide valuable data on exploring specific diagnostic tool for clinical tests.

## Supporting information

S1 Table The identified proteins from MS-based proteomic analysis. (PDF)
