# Supplementary material for: The use of in-strip digestion for fast proteomic analysis on tear fluid from dry eye patients
Source: PLoS One. 2018 Aug 3;13(8):e0200702. doi: 10.1371/journal.pone.0200702 (PMC6075744; doi:10.1371/journal.pone.0200702)
Supplement: S1 Table — (PDF) [file pone.0200702.s001.pdf]

S1 Table . The identified proteins from MS-based proteomic analysis.

Number	Accession	Description	Coverage	Peptides	Score Sequest HT
1	P02768	Serum albumin	73.2	52	543
2	P04264	Keratin 1	45.3	31	172.5
3	P35908	Keratin, type II cytkeletal 2 epidermal	38.3	22	93.1
4	P02787	Serotransferrin	31.3	21	72
5	A0A0K0K1H8	Epididymis secretory sperm binding protein Li 71p	30.5	21	72.5
6	P13645	Keratin, type I cytkeletal 10	47.9	20	99.5
7	P48668	keratin 6E (KRT6E	33	17	51.1
8	A0A161I202	Lactoferrin	30.9	16	49.1
9	P35527	Keratin, type I cytkeletal 9	28.1	13	62.9
10	P13647	Keratin, type II cytkeletal 5	22	13	38.5
11	P02533	Keratin, type I cytkeletal 14	27.5	12	37.6
12	P08779	Keratin, type I cytkeletal 16	25.8	11	38.4
13	E9KL23	Epididymis secretory sperm binding protein Li 44a	24.2	9	34.4
14	P63261	Actin, cytoplasmic 2	30.1	8	29.3
15	P02749	Apolipoprotein H (beta-2-glycoprotein I)	31.8	8	25.4
16	P00352	Retinal dehydrogenase 1	22.8	8	24.1
17	Q7Z351	Putative uncharacterized protein DKFZp686N02209	22.2	7	31.8
18	P06733	Alpha-enolase	24.4	6	21.5
19	P00738	Haptoglobin	15.8	6	18.4
20	Q04695	Keratin, type I cytkeletal 17	14.1	6	17.5
21	P12273	Prolactin-inducible protein	56.8	6	23.3
22	P01024	Complement C3	4.1	6	15.6
23	P10909	Clusterin	10	6	19.1
24	V9HWE9	Epididymis secretory protein Li 22	40	5	21
25	V9HWA9	Epididymis secretory sperm binding protein Li 62p	4	5	13.7
26	P25311	Zinc-alpha-2-glycoprotein	23.5	5	17.2
27	E7EX29	14-3-3 protein zeta/delta (Fragment)	21.5	4	10.5
28	A8K3K1	cDNA FLJ78096, highly similar to Homo sapiens actin, alpha, cardiac muscle (ACTC), mRNA	14.1	4	11.9
29	V9HWH1	Epididymis luminal protein 57	14.5	4	10.1
30	P02774-3	Isoform 3 of Vitamin D-binding protein	12.8	4	11.2
31	Q0IIN1	Keratin 77	5.4	4	29.4
32	P08727	Keratin, type I cytkeletal 19	9.3	4	11.2
33	Q86Y46	Keratin, type II cytkeletal 73	5.6	4	11.4

Number	Accession	Description	Coverage	Peptides	Score Sequest HT
34	P31025	Lipocalin-1	26.7	4	16.7
35	Q6N092	Putative uncharacterized protein DKFZp686K18196 (Fragment)	11.2	4	10
36	A0A024R5Z9	Pyruvate kinase	10.7	4	12
37	Q68CN4	Uncharacterized protein	13.2	4	16.7
38	H0Y7A7	Calmodulin (Fragment)	24.6	3	8.3
39	A8K9C4	Elongation factor 1-alpha	10.2	3	9
40	V9HW43	Epididymis secretory protein Li 102	28.3	3	13.8
41	P04406	Glyceraldehyde-3-phphate dehydrogenase	17	3	10.2
42	P02790	Hemopexin	10.4	3	9.4
43	Q6GMX4	IGL@ protein	14	3	9.7
44	P05109	Protein S100-A8	31.2	3	10
45	Q8TCD0	Uncharacterized protein	22.2	3	18.1
46	Q9GZZ8	Lacritin	10.5	3	12.2
47	P02763	Alpha-1-acid glycoprotein 1/oromucoid 1	11.4	2	9.6
48	P01023	Alpha-2-macroglobulin	1.8	2	5.2
49	B3KS79	cDNA FLJ35730 fis, clone TESTI2003131, highly similar to ALPHA-1-ANTICHYMOTRYPSIN	3.3	2	4.1
50	P02765	Alpha-2-HS-glycoprotein	7.9	2	8.3
51	A8K5A4	cDNA FLJ76826, highly similar to Homo sapiens ceruloplasmin (ferroxidase) (CP), mRNA	2.3	2	4.3
52	V9HWG3	Epididymis secretory protein Li 45	3.3	2	4.3
53	P61626	Lysozyme C	18.9	2	6.3
54	Q16378	Proline-rich protein 4	10.2	2	5.8
55	B2R4M6	Protein S100	24.6	2	20
56	P26447	Protein S100-A4	12.5	2	2.3
57	P06703	Protein S100-A6	15.2	2	2
58	P60174	Triephphate isomerase	9.8	2	6
59	E7EQ64	Trypsin-1	6.9	2	7.8
60	P04083	Annexin A1	9.5	2	2.8
61	P04792	Heat shock protein beta-1	9.8	2	5.4
62	Q9UGM3	Deleted in malignant brain tumors 1 protein	16	2	5.1
63	P04792	Heat shock protein beta-1	6.5	2	8.2
64	P01591	Immunoglobulin J chain	16.3	2	5.1
65	P01009	Serpin peptidase inhibitor 1	5.9	2	5.8
66	Q99935	Proline rich, lacrimal 1	7.6	2	7.3
67	P09211	Glutathione S-transferase pi 1	4.2	2	6.5
68	A5A3E0	POTE ankyrin domain family, member F	5.6	2	7.1

Number	Accession	Description	Coverage	Peptides	Score Sequest HT
69	Q08188	Transglutaminase 3	9.6	2	8.4
70	Q8NI51	CCCTC-binding factor (zinc finger protein)-like	5.6	2	5.7
71	V9GYM3	Apolipoprotein A-II	7.5	1	2.3
72	P02749	Beta-2-glycoprotein 1	4.9	1	3.2
73	P09467	Fructe-1,6-bisphphatase 1	4.7	1	2.4
74	Q59FR8	Galectin (Fragment)	4.3	1	2.4
75	P06396	Gelsolin	1.4	1	2.9
76	B2R4R0	Histone H4	11.7	1	2.3
77	Q5BKZ8	HSPA12B protein (Fragment)	1.3	1	2.4
78	P07355-2	Isoform 2 of Annexin A2	5	1	2.7
79	X6R8F3	Neutrophil gelatinase-associated lipocalin	10.5	1	2.8
80	A8K486	Peptidyl-prolyl cis-trans isomerase	7.3	1	2.9
81	E9PGN7	Plasma protease C1 inhibitor	3.1	1	2.9
82	P01833	Polymeric immunoglobulin receptor	2.5	1	5.3
83	Q7Z6P3	Ras-related protein Rab-44	2.8	1	4.9
84	Q65ZC9	Single-chain Fv (Fragment)	7.9	1	3.1
85	O75556	Secretoglobin, family 2A, member 1	12.2	1	7.7
86	Q4L180	Filamin A interacting protein 1-like	3.5	1	5.2
